# Benchmarking AI Chatbots for Maternal Lactation Support: A Cross-Platform Evaluation of Quality, Readability, and Clinical Accuracy

**DOI:** 10.3390/healthcare13141756

**Published:** 2025-07-20

**Authors:** İlke Özer Aslan, Mustafa Törehan Aslan

**Affiliations:** 1 Assisted Reproductive Technologies Unit, Acıbadem Maslak Hospital, Istanbul 34457, Türkiye; 2 Department of Neonatology, Koç University Hospital, Istanbul 34010, Türkiye; torehanaslan@yahoo.com

**Keywords:** artificial intelligence, breastfeeding, chatbot, clinical accuracy, patient education, lactation support, large language models

## Abstract

**Background and Objective:** Large language model (LLM)–based chatbots are increasingly utilized by postpartum individuals seeking guidance on breastfeeding. However, the chatbots’ content quality, readability, and alignment with clinical guidelines remain uncertain. This study was conducted to evaluate and compare the quality, readability, and factual accuracy of responses generated by three publicly accessible AI chatbots—ChatGPT-4o Pro, Gemini 2.5 Pro, and Copilot Pro—when prompted with common maternal questions related to breast-milk supply. **Methods:** Twenty frequently asked breastfeeding-related questions were submitted to each chatbot in separate sessions. The responses were paraphrased to enable standardized scoring and were then evaluated using three validated tools: ensuring quality information for patients (EQIP), the simple measure of gobbledygook (SMOG), and the global quality scale (GQS). Factual accuracy was benchmarked against WHO, ACOG, CDC, and NICE guidelines using a three-point rubric. Additional user experience metrics included response time, character count, content density, and structural formatting. Statistical comparisons were performed using the Kruskal–Wallis and Wilcoxon rank-sum tests with Bonferroni correction. **Results:** ChatGPT-4o Pro achieved the highest overall performance across all primary outcomes: EQIP score (85.7 ± 2.4%), SMOG score (9.78 ± 0.22), and GQS rating (4.55 ± 0.50), followed by Gemini 2.5 Pro and Copilot Pro (*p* < 0.001 for all comparisons). ChatGPT-4o Pro also demonstrated the highest factual alignment with clinical guidelines (95%), while Copilot showed more frequent omissions or simplifications. Differences in response time and formatting quality were statistically significant, although not always clinically meaningful. **Conclusions:** ChatGPT-4o Pro outperforms other chatbots in delivering structured, readable, and guideline-concordant breastfeeding information. However, substantial variability persists across the platforms, and none should be considered a substitute for professional guidance. Importantly, the phenomenon of AI hallucinations—where chatbots may generate factually incorrect or fabricated information—remains a critical risk that must be addressed to ensure safe integration into maternal health communication. Future efforts should focus on improving the transparency, accuracy, and multilingual reliability of AI chatbots to ensure their safe integration into maternal health communications.

## 1. Introduction

Breastfeeding is universally recognized as the optimal source of nutrition for infants, offering extensive benefits for both neonatal development and maternal health. The World Health Organization (WHO) recommends exclusive breastfeeding for the first six months of life, yet many mothers encounter challenges in maintaining an adequate breast-milk supply during this period. Globally, insufficient milk production remains one of the leading causes of early breastfeeding discontinuation, contributing to suboptimal infant growth, an increased risk of infections, and early transition to formula feeding [1].

To address these concerns, mothers frequently turn to online platforms and digital health tools in search of accessible, immediate, and personalized guidance on how to increase breast-milk production. In recent years, artificial intelligence (AI)-based chatbots—such as ChatGPT (OpenAI), Gemini (Google), and Copilot (Microsoft)—have emerged as widely available tools for generating health-related content in real time. These large language models (LLMs) are trained on massive corpora of online text and can simulate human-like conversation, enabling them to provide general information, advice, and educational materials on a wide range of medical topics.

Although some LLMs have demonstrated reasonable accuracy in fields like dermatology, oncology, and symptom triage [2,3,4,5,6], their application to maternal health remains comparatively underexplored. Recent reviews emphasize that while AI-based chatbots offer promise for augmenting patient education, their content often lacks sufficient validation, structure, and alignment with evidence-based recommendations [2,3]. Moreover, studies have shown that the tone and phrasing used by chatbots can sometimes lead to overconfidence in AI-generated guidance, especially in emotionally charged health decisions such as breastfeeding [7,8].

Despite emerging interest in AI’s role in maternal care [2,3], to our knowledge, there has been no structured comparison of the quality, readability, and clinical accuracy of chatbot-generated responses that are focused specifically on lactation support. This gap is critical, as breastfeeding guidance is often sought by vulnerable subgroups—first-time mothers, women with premature infants, or individuals experiencing postpartum stress—at a time when misinformation may negatively affect maternal confidence and infant health outcomes.

Given the widespread reliance on digital tools, it is essential to determine whether chatbot-generated breastfeeding advice is consistent with the best available clinical evidence. International clinical guidelines from leading health authorities—including the World Health Organization (WHO), the American College of Obstetricians and Gynecologists (ACOG), the Centers for Disease Control and Prevention (CDC), and the UK’s National Institute for Health and Care Excellence (NICE)—offer standardized, evidence-based recommendations on breastfeeding techniques, support mechanisms, and lactation management strategy triage [9,10,11,12]. National guidelines were not included as this study aimed to benchmark chatbots against globally recognized standards to ensure cross-country comparability. These guidelines provide a robust benchmark for evaluating the medical validity of AI-generated content and are globally recognized for their comprehensive scope, accessibility, and applicability across various healthcare systems.

The current study aims to systematically evaluate the quality, readability, and factual alignment of AI chatbot responses to common maternal questions related to breast-milk supply. Specifically, we compare three widely-used platforms—ChatGPT-4, Gemini, and Copilot—across multiple validated metrics, including the ensuring quality information for patients (EQIP) tool, the simple measure of gobbledygook (SMOG) index, and the global quality scale (GQS), while assessing guideline concordance using international breastfeeding standards [13,14,15,16]. By identifying the strengths and weaknesses of these platforms in the context of maternal digital communication, we hope to inform clinicians, public health practitioners, and the developers of AI tools about the potential for and limitations of LLMs in the context of breastfeeding education.

## 2. Methods

### 2.1. Study Design and Overview

This study was designed as a structured, cross-platform benchmarking evaluation of large language model (LLM)–based AI chatbots in the context of maternal lactation support. We used the latest public versions of each chatbot as of 18 May 2025: ChatGPT-4o Pro (OpenAI), Gemini-2.5 Pro (Google), and Copilot Pro (Microsoft, powered by GPT-4 Turbo). The comparison was conducted using a blinded, parallel-query model to reduce platform bias.

### 2.2. Setting and Participants

As this study did not involve direct patient interaction or identifiable health information, no human participants were enrolled. Instead, we simulated real-world use scenarios by selecting 20 frequently asked questions derived from public sources. All chatbot queries were submitted by clinicians via official web interfaces under identical browser conditions.

### 2.3. Materials and Instruments

Three validated tools were used for evaluation. Ensuring quality information for patients (EQIP) comprises a 20-item checklist that assesses the accuracy, completeness, structure, and source attribution of health information. The simple measure of gobbledygook (SMOG) is a readability index estimating the education level required to understand the material. To assess the potential impact of paraphrasing on readability, a sensitivity analysis was performed by calculating SMOG scores for the original, unedited chatbot responses. These results are presented in Supplementary Table S3. The global quality scale (GQS) comprises a five-point Likert scale rating the clarity and usefulness of content from a layperson’s perspective.

Each chatbot response was paraphrased to standardize the language, eliminate branding, and ensure comparable scoring. The paraphrased outputs preserved the original meaning and were independently reviewed by two physicians (a neonatologist and an obstetrician–gynecologist). In addition, four exploratory user experience (UX) metrics were recorded: response time (in seconds), character count, content density (clinical concepts per 100 words), and the structural formatting score (use of bullet points, headings, and paragraphing). The definitions and procedures for each metric are detailed below.

### 2.4. Procedures

*Question Selection*: The 20 questions were selected using a triangulated approach: (1) a thematic analysis of discussions from parenting forums (e.g., Reddit and BabyCenter), (2) trending keywords from Google Trends, and (3) clinician judgments from outpatient lactation clinics. Candidate questions were reviewed and refined for clarity, clinical relevance, and representativeness of maternal language. The 20 questions were selected using triangulated methods to reflect real-world user concerns and ensure thematic representativeness. We did not perform formal pilot testing, but inter-rater consensus was used to finalize each question’s phrasing. The final list is provided in Appendix A.

*Querying Process:* Each question was submitted in an independent chatbot session to prevent learning carryover. Prompts were entered in a neutral tone without follow-up. All interactions occurred on 18 May 2025. Responses were manually paraphrased to achieve a uniform structure and tone prior to scoring, in order to eliminate brand language, inconsistent formatting, and redundant phrases. While this may have altered the surface-level readability (as measured by SMOG), it also enabled fairer EQIP and GQS evaluation. This methodological choice is acknowledged as a limitation and should be considered in interpreting the readability results.

### 2.5. Outcomes and Variables

*Primary outcomes included:* Quality of information (EQIP% score), readability (SMOG score), and perceived usefulness (GQS rating).

*Secondary/exploratory outcomes:* The responses’ factual accuracy was compared with guideline-based reference answers (scored on a 3-point scale: 0 = inaccurate, 1 = partially accurate, and 2 = fully concordant). A detailed side-by-side comparison of guideline-based reference answers and the chatbot-generated responses for each question is provided in Supplementary File S1. Response time was measured with a stopwatch and validated via browser console timestamps. Content density was defined as the number of distinct evidence-based clinical concepts per 100 words. Concepts were pre-coded by two clinicians using a consensus-based reference list. Inter-coder agreement (Cohen’s κ) was calculated and exceeded 0.80. Formatting quality was rated based on the presence of paragraph segmentation, bulleted lists, and visual structure. No single primary outcome was pre-defined, as the aim was to achieve multidimensional benchmarking. However, EQIP was emphasized as the most clinically relevant metric.

### 2.6. Statistical Analysis

Descriptive statistics (mean ± SD, median, and IQR) were computed. Kruskal–Wallis tests were used to compare groups across non-parametric and ordinal data. Post hoc pairwise comparisons employed Wilcoxon rank-sum tests with Bonferroni correction. Effect sizes (η^2^ for Kruskal–Wallis; r for Wilcoxon) were also calculated to contextualize any differences. Inter-rater reliability was assessed using Cohen’s kappa for EQIP, SMOG, GQS, and content density; *p*-values of < 0.05 were considered significant. Analyses were performed in Jamovi v2.4 and Python 3.11 (pandas and scipy).

### 2.7. Ethical Approval

This study did not involve human subjects, patients, or identifiable personal data; therefore, it was exempt from ethical review according to the current regulations.

## 3. Results

A total of 20 standardized, mother-centered questions related to strategies for increasing breast milk production were submitted to three AI-based chatbots: ChatGPT-4, Gemini, and Copilot. Each response was independently evaluated by two expert reviewers using the EQIP, SMOG, and GQS metrics. ChatGPT-4 consistently demonstrated superior performance across all three primary metrics, followed by Gemini and Copilot. Reviewer agreement across the domains was high (Cohen’s κ = 0.81–0.88), supporting the objectivity of this scoring system.

### 3.1. Quality of Responses (EQIP Scores)

ChatGPT achieved the highest mean EQIP score (85.7 ± 2.4%, 95% CI: 84.6–86.8), significantly outperforming Gemini (83.9 ± 1.7%, 95% CI: 83.1–84.7) and Copilot (80.1 ± 1.3%, 95% CI: 79.5–80.7) (*p* < 0.001). Pairwise Wilcoxon tests confirmed ChatGPT’s superiority over both platforms. Table 1 provides a summary of group-level EQIP, SMOG, and GQS scores, including standard deviations and 95% confidence intervals, as well as their significance levels. The full per-question EQIP matrix (20 × 3) is available in Supplementary Table S1 for clarity.

### 3.2. Readability Outcomes (SMOG Index)

ChatGPT responses were the easiest to read, with a mean SMOG score of 9.78 ± 0.22 (95% CI: 9.67–9.89). Gemini responses were slightly more complex (10.13 ± 0.10, 95% CI: 10.08–10.18), and Copilot scored highest (i.e., was hardest to read) at 10.61 ± 0.13 (95% CI: 10.54–10.68). All differences were statistically significant (*p* < 0.001), as confirmed by pairwise comparisons. A sensitivity analysis using the original chatbot outputs showed slightly higher SMOG scores across all platforms (Supplementary Table S3), reflecting the minor simplifications introduced during paraphrasing. ChatGPT’s readability corresponded to a high-school level of understanding, while Copilot’s responses required a near-college level of comprehension.

### 3.3. Perceived Usefulness (GQS Ratings)

ChatGPT achieved the highest mean GQS rating (4.55 ± 0.50, 95% CI: 4.32–4.78), followed by Gemini (4.40 ± 0.49, 95% CI: 4.18–4.62). Copilot trailed behind with a mean score of 3.45 ± 0.50 (95% CI: 3.22–3.68). The differences across platforms were statistically significant (*p* < 0.001), underscoring ChatGPT’s perceived superior usefulness.

### 3.4. Factual Accuracy Relative to Guidelines

**Figure 1:**
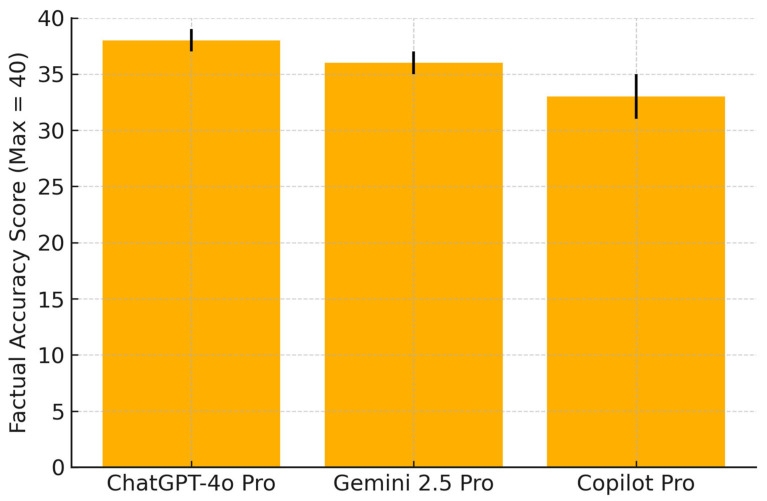
Total factual accuracy scores according to chatbot (max = 40).

Factual alignment with international guidelines was highest for ChatGPT (38/40 correct responses), followed by Gemini (36/40) and Copilot (33/40). Figure 1 illustrates these comparative totals. Individual item-level scores are detailed in Supplementary Table S2.

Factual accuracy was evaluated by comparing each chatbot’s response to 20 standardized breastfeeding-related questions against reference answers derived from WHO, ACOG, CDC, and NICE guidelines. The responses were scored by two independent clinicians using a 3-point rubric: 0 = inaccurate, 1 = partially accurate, 2 = fully concordant. The maximum possible score was 40. Error bars represent the standard deviations. ChatGPT-4o demonstrated the highest overall factual alignment, followed by Gemini 2.5 Pro and Copilot Pro.

### 3.5. User Experience Metrics

User experience parameters were analyzed to evaluate usability, including response time, response length (character count), content density, and formatting structure. The results are summarized in Table 2.

Although all differences were statistically significant, the response time gap (e.g., 3.9 vs. 5.1 s) is likely of limited clinical relevance and should be interpreted accordingly. In contrast, the density of clinically meaningful concepts and structured formatting likely contribute more directly to user comprehension and adherence.

### 3.6. Inter-Rater Agreement and Reliability

Cohen’s kappa coefficients were high for all metrics: EQIP (κ = 0.83), SMOG (κ = 0.81), and GQS (κ = 0.88), indicating substantial to near-perfect agreement. Paired *t*-tests revealed no significant differences between the reviewers (*p* > 0.3 for all domains).

## 4. Discussion

The findings of this study provide a structured, quantitative comparison of AI chatbot-generated responses to mother-centered breastfeeding questions. ChatGPT-4 consistently outperformed Gemini and Copilot in terms of information quality, readability, and clinical accuracy. These results align with prior studies suggesting that GPT-based platforms are better equipped to synthesize coherent and medically plausible outputs compared to other proprietary architectures [4,17]. Although multiple recent evaluations have assessed LLMs in maternal or perinatal health domains [2,3,8], this study is among the first to offer a cross-platform, head-to-head benchmarking of chatbot outputs using standardized tools specific to patient education and guideline concordance. Nonetheless, we recognize that the novelty claim must be interpreted with caution, given the rapidly evolving nature of LLM evaluation literature. This study was designed as an exploratory benchmarking analysis to provide a preliminary assessment of large language model (LLM)–based chatbots in the context of maternal lactation support. The selection of 20 thematically representative, frequently asked questions was guided by a triangulated approach (parenting forums, Google Trends, and clinician expertise) to simulate common real-world maternal concerns while maintaining methodological feasibility. This thematic diversity enhances ecological validity and provides valuable early insights into chatbot performance, helping to identify key areas for future large-scale investigations.

A key strength of our methodology lies in the multidimensional scoring approach—combining content quality (EQIP), readability (SMOG), perceived usefulness (GQS), and factual alignment with global breastfeeding guidelines. These tools complement each other in capturing both structural rigor and real-world utility. For instance, while EQIP evaluates the completeness and organization of information, SMOG quantifies linguistic accessibility, and the GQS incorporates subjective perceptions of clarity and helpfulness.

One important observation was the variability found in guideline adherence. While ChatGPT aligned well with WHO, ACOG, CDC, and NICE recommendations in most cases, other platforms occasionally failed to communicate safety limitations or misrepresented evidence strength. For example, in response to Question 6 (“Do lactation teas or cookies really work?”), Copilot did not mention the lack of strong evidence and omitted safety caveats—potentially misleading users into over-reliance on unproven remedies. Such subtle misinformation may delay appropriate interventions or erode maternal confidence, particularly among first-time mothers with low health literacy.

This concern is supported by prior literature showing that inaccurate or overly simplified breastfeeding advice can contribute to early weaning, unnecessary formula supplementation, or delayed lactation consultation [18]. In neonatal and preterm populations, where breastfeeding is critical for positive immunological and neurodevelopmental outcomes, such consequences may be clinically significant. Although this study benchmarked factual accuracy against international clinical guidelines, we acknowledge the previous phrasing in our limitations section suggesting that “accuracy was not directly evaluated.” We have amended this to clarify that factual alignment was indeed assessed, using a structured 3-point rubric and expert consensus. The usability analysis revealed that beyond structural accuracy, elements such as concise formatting, content density, and immediate responsiveness strongly influence perceived trust and engagement—especially in postpartum individuals experiencing an emotional and cognitive burden [19,20,21]. While ChatGPT scored best in all these domains, we emphasize that none of the evaluated platforms should be considered standalone sources of lactation advice.

Our results highlight the persistent limitations of LLMs, particularly the black-box nature of their decision-making processes. These systems generate responses without citing sources or conveying uncertainty levels, which may foster overconfidence in end-users. A further critical issue is the phenomenon of ‘AI hallucinations,’ where chatbots may produce factually incorrect, misleading, or fabricated information with high confidence [22,23,24]. Such outputs could undermine maternal confidence, delay appropriate interventions, and pose safety risks in sensitive contexts like breastfeeding guidance. Strategies for mitigating hallucinations—such as fine-tuning with curated medical datasets, implementing real-time validation protocols, and integrating explainability tools—should be prioritized in future models. We strongly recommend the development of transparent ethical frameworks to govern LLM use in patient-facing healthcare applications, including disclaimers, content validation protocols, and risk-based triaging mechanisms.

This study was conducted solely in English, which limits its generalizability to non-English-speaking populations. Given the linguistic diversity of maternal populations, future work should prioritize the multilingual validation of chatbot responses, particularly in low-resource settings. Regional or culturally tailored guidance may differ from global norms, and English-only evaluations may overlook important equity considerations.

## 5. Conclusions

This study provides one of the first structured, cross-platform evaluations of AI chatbot performance in the context of maternal lactation support. Among the platforms analyzed, ChatGPT-4 consistently outperformed Gemini and Copilot in terms of information quality, readability, and clinical accuracy. However, substantial variability across platforms and question types highlights the current limitations of large language models in delivering standardized, evidence-aligned health guidance. Inaccurate or overly simplified outputs, even if rare, may carry disproportionate risks in sensitive domains such as perinatal and breastfeeding care. These findings reinforce the necessity of expert oversight, transparent sourcing, and dynamic content validation before LLMs can be safely integrated into maternal healthcare delivery. Until such safeguards are established, the clinical role of AI chatbots in breastfeeding support should remain strictly adjunctive rather than authoritative. We urge policymakers, technology developers, and healthcare systems to prioritize the creation of ethical frameworks, fine-tuning strategies using validated guideline corpora, and multilingual expansion of chatbot models to ensure equitable, safe, and culturally sensitive maternal health communication. Further research is needed to assess real-world usage patterns, behavioral responses, and the long-term outcomes of AI-guided lactation advice across diverse populations.

### 5.1. Strengths, Limitations, and Declarations

This study offers a timely and methodologically rigorous evaluation of AI-generated responses to mother-centered breastfeeding questions, comparing the performance of three prominent large language model–based chatbots: ChatGPT-4, Gemini, and Copilot. By employing a standardized set of 20 questions derived from real-world maternal health concerns and applying three validated assessment tools—EQIP for content quality, SMOG for readability, and GQS for overall utility—this study provides a comprehensive, multidimensional assessment of chatbot output. The inclusion of blinded, independent evaluations by two domain experts and the demonstration of substantial to near-perfect inter-rater agreement lend strong internal validity and reproducibility to the findings. However, several limitations should be acknowledged. First, the research was conducted exclusively in English, which limits its generalizability to non-English-speaking populations or culturally specific maternal care practices. Future studies should evaluate multilingual chatbot outputs and assess consistency across different linguistic and health literacy backgrounds. Second, while we benchmarked factual accuracy against international guidelines (WHO, ACOG, CDC, NICE), domestic recommendations and real-time patient use scenarios were not evaluated. Third, the evaluation was based on a relatively small set of 20 standardized questions. However, this sample was intentionally selected using a triangulated approach (parenting forums, Google Trends, and clinician expertise) to ensure thematic representativeness across physiological, behavioral, and external factors. This breadth reduces the likelihood of bias from a single-type question set, though it still limits the breadth of benchmarking. Future research should expand to larger, more diverse question pools and longitudinal designs to enhance generalizability. Although numerous alternative AI platforms such as Med-PaLM 2, Claude, and Perplexity have shown promise in medical information generation, we deliberately focused on ChatGPT-4o Pro, Gemini 2.5 Pro, and Copilot Pro due to their wide public accessibility, cross-domain usage, and general popularity among lay users. These models represent mainstream tools that are currently available to postpartum individuals seeking self-guided health advice. Future research should explore comparisons involving clinically fine-tuned or specialty-specific models, especially in controlled healthcare environments. The simulated questions, although derived from real-world sources, may not fully reflect the emotional nuances or urgency of live user interactions. Third, all chatbot responses were analyzed at a single time point (May 2025) and may not reflect future model updates. Similarly, SMOG readability scoring was performed on paraphrased outputs to enable standardized comparison, which may not fully mirror actual user-facing content. Despite these limitations, the findings offer a timely and methodologically rigorous foundation for evaluating generative AI in maternal health communications.

### 5.2. Future Directions

From a clinical perspective, LLMs may serve as adjunctive tools in patient education and triage, especially when designed to supplement—not replace—evidence-based counseling. Future research should explore their use in real-time clinical environments, assessing how users interpret, trust, and act upon AI-generated advice. To improve safety and reliability, next-generation chatbots should incorporate transparent source attribution, explainability features, and mechanisms for continuous user feedback. Equally important is the validation of chatbot responses across multiple languages and cultural contexts to ensure equitable access to high-quality maternal guidance worldwide. Ultimately, the integration of generative AI into perinatal care should be guided by robust ethical frameworks, interdisciplinary oversight, and ongoing evaluation of its impact on health outcomes, provider trust, and patient empowerment.

## Figures and Tables

**Table 1:** Mean EQIP, SMOG, and GQS Scores (±SD, 95% CI) for three AI chatbots.

AI Chatbots	Mean EQIP (%) ± SD (95% CI)	Mean SMOG ± SD (95% CI)	Mean GQS ± SD (95% CI)
ChatGPT-4o Pro	85.7 ± 2.4 (84.6–86.8)	9.78 ± 0.22 (9.67–9.89)	4.55 ± 0.50 (4.32–4.78)
Gemini 2.5 Pro	83.9 ± 1.7 (83.1–84.7)	10.13 ± 0.10 (10.08–10.18)	4.40 ± 0.49 (4.18–4.62)
Copilot Pro	80.1 ± 1.3 (79.5–80.7)	10.61 ± 0.13 (10.54–10.68)	3.45 ± 0.50 (3.22–3.68)

EQIP, Ensuring quality information for patients; SMOG, simple measure of gobbledygook; GQS, global quality scale; SD, standard deviation; CI, confidence interval.

**Table 2:** User experience metrics of AI chatbots in maternal lactation queries—comparative analysis.

Metric	ChatGPT-4o Pro (Mean ± Standard Deviation)	Gemini 2.5 Pro (Mean ± Standard Deviation)	Copilot Pro (Mean ± Standard Deviation)	*p*
Response Time (s)	4.2 ± 0.5	5.1 ± 0.6	3.9 ± 0.4	0.017
Character Count	987 ± 45	1045 ± 52	1103 ± 61	0.028
Content Density (/100 words)	4.8 ± 0.3	4.5 ± 0.2	3.9 ± 0.4	<0.001
Structured Formatting (%)	90	65	45	<0.001

## Data Availability

All data supporting the findings of this study are included in the main text and Supplementary Materials. The dataset consists of AI-generated responses to standardized breastfeeding-related questions, which were manually paraphrased and evaluated using validated assessment tools. No new or sensitive datasets were created or collected during the study. Additional information can be made available upon reasonable request from the corresponding author.

## Supplementary Materials

The following supporting information can be downloaded at: https://www.mdpi.com/article/10.3390/healthcare13141756/s1, Supplementary Table S1: Detailed EQIP, SMOG, GQS for all 20 questions; Supplementary Table S2: Factual accuracy per question vs guideline; Supplementary Table S3. SMOG Readability Scores for Original Chatbot Responsesy. File S1: Per-question comparison of guideline-based answers and chatbot responses.

## Appendix A

Mother-centered questions on increasing breast-milk supply.

**Table:**

No	Questions
1.	What can I eat or drink to make more breast milk?
2.	How can I tell if I’m making enough milk for my baby?
3.	Does drinking more water help me produce more milk?
4.	Will pumping after breastfeeding help me make more milk?
5.	What natural ways can I try to boost my breast-milk supply?
6.	Do lactation teas or cookies really work?
7.	Can stress or anxiety lower my milk production?
8.	How often should I breastfeed to increase milk?
9.	Is it okay to use both breasts at each feeding to make more milk?
10.	Can I still make enough milk if I had a C-section?
11.	Do I need to wake my baby at night to maintain breast-milk supply?
12.	Will taking vitamins or supplements help with milk production?
13.	Can using a warm compress help improve milk flow or supply?
14.	Should I avoid any foods if my breast-milk supply is low?
15.	How long does it take to see an increase in milk after trying new methods?
16.	Will using a breast pump regularly increase my supply?
17.	What are the signs of low breast-milk supply?
18.	Does breastfeeding while lying down affect milk production?
19.	Can I continue to breastfeed if I return to work and want to maintain my supply?
20.	When should I see a lactation consultant if my milk seems low?
