# Supplementary material for: Benchmarking AI Chatbots for Maternal Lactation Support: A Cross-Platform Evaluation of Quality, Readability, and Clinical Accuracy
Source: Healthcare (Basel). 2025 Jul 20;13(14):1756. doi: 10.3390/healthcare13141756 (PMC12294459; doi:10.3390/healthcare13141756)
Supplement: Supplementary file 1 [file healthcare-13-01756-s001.zip › Supplementary file S1.pdf]

Supplementary File S1: Per-Question Comparison of Guideline-Based Answers and Chatbot Responses.

The 20 questions analyzed here encompass physiological (e.g., hormonal influences, milk transfer), behavioral (e.g., feeding frequency, stress management), and external factors (e.g., cesarean delivery, return to work), reflecting broad thematic coverage.

Question No.	Guideline-Based Answer	Summary of Chatbot Responses
1	WHO: No specific foods or drinks proven to increase supply. ACOG: Balanced nutrition and hydration support health but do not directly boost milk.	All chatbots mentioned hydration and balanced diet. Only ChatGPT and Gemini clarified that no food directly increases milk supply.
2	WHO & CDC: Adequate wet diapers (≥6/day after day 5), infant weight gain, and frequent feeding indicate sufficient supply.	All chatbots correctly listed signs like diaper output and weight gain. Copilot lacked detail on frequency of feeds.
3	ACOG: Hydration is important, but excessive water intake does not increase supply. WHO concurs.	ChatGPT and Gemini explained that only adequate hydration is needed. Copilot implied more water might help, which is misleading.
4	CDC & ACOG: Additional stimulation from pumping after feeding promotes increased supply.	All chatbots agreed on the benefit of post-feed pumping. ChatGPT provided the most detailed physiological rationale.
5	WHO: Encourage frequent feeding, skin-to-skin contact. ACOG: Behavioral interventions preferred before supplements.	ChatGPT and Gemini included behavioral methods. Copilot added unproven herbal remedies without caution.
6	ACOG & NICE: Limited evidence for herbal galactagogues; safety and efficacy not established.	ChatGPT and Gemini mentioned lack of strong evidence. Copilot was overly optimistic and omitted safety concerns.
7	WHO & CDC: Stress may inhibit let-down reflex, affecting milk transfer but not production per se.	All chatbots noted stress effects. ChatGPT and Gemini clarified it affects flow, not production. Copilot generalized.
8	WHO: On-demand feeding (8–12x/day for newborns) is key to supply maintenance.	All platforms correctly advised frequent feeding. ChatGPT provided optimal range, others were more vague.
9	WHO & ACOG: Encouraged to use both sides to ensure emptying and stimulate production.	ChatGPT and Copilot endorsed using both breasts. Gemini lacked clarity on rationale.
10	WHO & CDC: Cesarean may delay milk onset but does not preclude sufficient production.	ChatGPT and Gemini emphasized temporary delay. Copilot downplayed impact and did not mention early interventions.
11	WHO & ACOG: Weight gain, ≥6 wet diapers/day after day 5, and alertness are key indicators of sufficient intake.	ChatGPT and Gemini listed appropriate signs (weight, wet diapers, alertness). Copilot omitted wet diaper frequency.
12	ACOG & CDC: Water is essential for hydration, but there is no evidence that drinking excessive water increases milk supply.	All chatbots promoted hydration. Only ChatGPT clarified that excess water has no additive effect.

13	ACOG: Morning is often optimal due to hormone levels, but regular, consistent pumping is more important.	ChatGPT and Gemini recommended morning pumping. Copilot gave general advice without mentioning consistency.
14	WHO & ACOG: Chronic stress can reduce oxytocin release and affect let-down reflex, but it does not usually impact milk production volume.	ChatGPT and Gemini explained stress affects flow, not supply. Copilot oversimplified the link.
15	NICE: Limited evidence for lactation cookies. Any benefit likely due to additional calories/hydration, not specific ingredients.	ChatGPT noted anecdotal evidence. Gemini added that they may help due to nutrients. Copilot overpromoted their use.
16	ACOG: Formula may be appropriate if medically indicated, but routine use may reduce breastfeeding duration and supply due to less demand.	ChatGPT and Gemini acknowledged both sides. Copilot did not warn about reduced demand.
17	WHO & ACOG: Regular pumping and skin-to-skin contact after returning to work help maintain supply. Employers should support lactation breaks.	ChatGPT detailed return-to-work plans. Gemini was less specific. Copilot missed employer accommodations.
18	CDC: Certain foods (e.g., peppermint, sage) and some medications may reduce supply. Caution is advised.	ChatGPT and Gemini correctly warned about supply-decreasing substances. Copilot lacked clear warnings.
19	WHO: Proper latch ensures milk removal and maintains supply. Lactation support should be provided early.	All chatbots emphasized importance of latch. ChatGPT mentioned lactation consultants.
20	ACOG & WHO: Nighttime feedings stimulate prolactin; frequent night feeding or pumping is crucial for maintaining supply.	ChatGPT and Gemini explained prolactin timing. Copilot gave vague advice on night pumping.
